# Users’ Perceptions of Access to and Quality of Public Health Services in Brazil: A Cross-Sectional Study in Metropolitan Rio de Janeiro, Including Pharmaceutical Services

**DOI:** 10.3390/ijerph22060967

**Published:** 2025-06-19

**Authors:** Mariana Crespo Raimundo, Edna Afonso Reis, Igor Fradique Leandro Ferraz, Carlos Podalirio Borges de Almeida, Brian Godman, Stephen M. Campbell, Johanna C. Meyer, Isabella Piassi Dias Godói

**Affiliations:** 1 Institute of Pharmaceutical Sciences, Federal University of Rio de Janeiro, Avenida Aluízio da Silva Gomes, 50, Granja dos Cavaleiros, Macaé CEP 27930-560, Rio de Janeiro, Brazil; maricrespo.r2@gmail.com (M.C.R.); igrferraz@gmail.com (I.F.L.F.); 2 Department of Statistics, Exact Sciences Institute, Federal University of Minas Gerais, Avenida Antônio Carlos, 6627, Campus Pampulha, Belo Horizonte CEP 31270-901, Minas Gerais, Brazil; ednareis@gmail.com; 3 Health Technology Assessment Center—Management, Economics, Health Education and Pharmaceutical Services (GEESFAR/NATS/UFRJ) of the Federal University of Rio de Janeiro, Rio de Janeiro CEP 27930-560, Rio de Janeiro, Brazil; 4 Public Health Faculty, Institute of Health and Biological Studies, Federal University of Sul e Sudeste do Pará, Avenida dos Ipês, s/n, Cidade Universitária, Loteamento Cidade Jardim, Marabá CEP 68508-970, Pará, Brazil; carlos.almeida@unifesspa.edu.br; 5 Department of Global Health and Social Medicine, Harvard Medical School, Boston, MA 02115, USA; 6 Strathclyde Institute of Pharmacy and Biomedical Sciences, University of Strathclyde, Glasgow G1 1XQ, UK; brian.godman@strath.ac.uk; 7 Department of Public Health Pharmacy and Management, School of Pharmacy, Sefako Makgatho Health Sciences University, Ga-Rankuwa, Pretoria 0208, South Africa; stephen.campbell@manchester.ac.uk (S.M.C.); hannelie.meyer@smu.ac.za (J.C.M.); 8 Antibiotic Policy Group, Institute for Infection and Immunity, City St. George’s, University of London, London SW17 0RE, UK; 9 School of Health Sciences, University of Manchester, Manchester M13 9PL, UK; 10 South African Vaccination and Immunisation Centre, Sefako Makgatho Health Sciences University, Ga-Rankuwa, Pretoria 0208, South Africa

**Keywords:** Brazil, access, quality, health services, users, public health, health management

## Abstract

Background: This study evaluates one of the five regions of the state of Rio de Janeiro, Brazil, as part of a broader research project examining users’ perceptions of the Unified Health System (SUS), which has already generated publications in previous phases. The aim was to assess users’ perceptions of the SUS regarding access to and the quality of public health services, including pharmaceutical services, in the Metropolitan Region of Rio de Janeiro State. Method: A cross-sectional study was conducted between January and August 2024 with 200 participants, using a 66-item survey addressing access to and the quality of SUS services, appointment scheduling, medication acquisition, and the pharmacist’s role. Associations between variables were investigated using the Pearson Chi-Square Test in R software. Results: Frequent SUS users rated access as very good/good (*p* = 0.002) and overall quality as very good/good (*p* = 0.045). Reported challenges included the need for improved infrastructure (48.5%), better professional qualifications (30.6%), and easier access to medicines (16.8%). Higher ratings were given by those who used the SUS more frequently, and, in general, there was a tendency for participants with lower socioeconomic conditions to provide more favorable assessments of access to public health services (*p* = 0.024). Conclusions: A universal health system should cover diverse regions with unique needs. However, 49.4% of participants stated they never received information on how to store their medicines, and 42.3% reported never encountering a pharmacist in public pharmacies. Further ongoing studies assessing user perceptions are essential to ensure users play a central role in health decision-making, contributing to the system’s strengthening and improvement.

## 1. Introduction

With the 1988 Brazilian Federal Constitution, Article 196, universal healthcare became a right for all citizens of Brazil and a duty of the State to provide healthcare [1], under a Health Users’ Rights Charter [2,3], within a Unified Health System (SUS) [2]. This public and complex system aims to provide universal, free, and high-quality access to health services for the entire Brazilian population, with a focus on health promotion, protection, and recovery. Over the years, the SUS has faced challenges and continued to progress in order to keep delivering quality healthcare to the population [4]. Notably, its core principles include universality, comprehensive care, and the regionalization of health services and actions [3].

Within the Constitutional Amendment of 2000 [5], resources for healthcare services are allocated at three levels of government: federal, states, and the municipalities. Based on tax collection, the federal union, states, and municipalities deliver a series of actions and services in a decentralized and hierarchical manner across different levels of complexity, i.e., primary, secondary, and tertiary care [6,7,8]. In general, less complex services are managed and provided at the municipal (primary care) level. The services include medical consultations and the storage and administration of immunobiologicals. Meanwhile, more complex services that typically require greater coordination and agreements between states and the union, such as performing transplants and surgeries [6,7,8], are delivered by the state or federal services.

The Family Health Strategy in 1994 prioritized the healthcare system and service delivery in Brazil based on primary healthcare (PHC) [9] within each municipality. Since this reorientation, there have been a number of population health improvements including reduced infant mortality and preventable hospitalizations [10,11]. PHC is considered by the World Health Organization (WHO) as the foundational cornerstone for a health system [12]. Hence, when accessible, quality PHC brings overall benefits to the population [11].

The provision of PHC is primarily organized in facilities known as basic health units and/or family health strategies. PHC teams consist of health professionals from diverse fields and categories (eMulti) working collaboratively with an integrated approach within the same population and territory [13]. This multidisciplinary team must include doctors, nurses, and community health agents (a health professional who works in health promotion and disease prevention, and who, in most cases, does not hold a higher education degree), while other professionals (e.g., pharmacists) may join these teams depending on municipal arrangements [14]. In this context, pharmacists have not contributed to, or been seen formally, as part of PHC in most municipalities in Brazil. It is important to emphasize that municipal administrations are primarily responsible for organizing and hiring professionals to work in PHC. However, data on the number and identification of municipalities, by state and across Brazil, that includes pharmacists as members of multidisciplinary teams at this level of care are not easily accessible or transparent.

Pharmacists are often seen performing administrative tasks, ensuring the necessary supply of resources in health units, and managing medication dispensing units [15]. Pharmaceutical care services, which involve an individualized and patient-centered process, including the provision of guidance and monitoring of medication use by a pharmacist are, unfortunately, currently not a reality in most Brazilian municipalities within the primary health care (PHC) setting. Pharmacists, however, undertake crucial roles in health promotion within the SUS, ensuring quality pharmacotherapy through the guidance and follow-up of patients [13,16]. Whilst the SUS provides free medicines to the population in its basic, strategic, and specialized component [17,18,19,20,21,22,23,24,25,26], and has programs in place to strengthen the pharmaceutical sector, most municipalities in the country still face challenges in accessing these medicines and ensuring adequate pharmaceutical services [15,16,27,28].

There are more than 300,000 professionals working as pharmacists in Brazil [29], with a rate of 2.02 pharmacists per 10,000 inhabitants in the PHC setting. The Southeast region has the highest rate of pharmacists second only to the South region with 2.11, the Midwest with 2.08, the North with 1.51, with the Northeast having the lowest rate of 1.18 pharmacists per 10,000 inhabitants [30]. In 2017, on average, pharmacists worked in 14.7% of basic health units within PHCs in Brazil. The presence of pharmacists in PHC is higher in smaller municipalities with a higher Municipal Human Development Index, particularly in the South and Southeast regions [30]. Pharmacists are less prevalent in cities with populations of between 20,000 and 100,000 inhabitants [31]. This reflects the fact that pharmacists are not mandatory members of family health teams in many municipalities [13,32,33], with many municipalities lacking pharmacists in basic health units and family health programs [31].

A study by the National Health Confederation revealed that approximately 56.5% of private hospitals provide care through the SUS, with 36.0% located in municipalities with over 500,000 inhabitants and only 13.0% in municipalities with up to 20,000 inhabitants [34]. In addition, despite offering free health services to its population through the SUS, approximately 24.6% of Brazilians also have private health plans, which can offer quicker surgery and consultations with specialists. However, citizens with private health coverage may still access SUS services, according to the National Supplementary Health Agency (ANS) [35].

While universal access to health services is a principle of the SUS, access to quality healthcare should go beyond physical access to the healthcare system and consider timely and routine availability based on responding to need, within available funds [36,37]. Regions with greater socioeconomic development, such as the Metropolitan area of Rio de Janeiro, also serve patients from neighboring regions in search of healthcare and access to health services [38,39]. Despite this, the Metropolitan portion also faces inequalities among the municipalities in its territory in areas such as healthcare, security, basic sanitation, employment, and education [40]. Given the aspects mentioned above, and the scarcity of studies evaluating this region, the importance of conducting research aimed at understanding the perceptions and demands of SUS users in this area is underscored.

According to Law 8142 of 1990, social participation is one of the main principles of the SUS [41]. Despite the creation of Health Councils and Health Conferences, this remains a challenging scenario in Brazil [42,43,44]. Consequently, the implementation of strategies that value the perceptions and experiences of discrete communities with varying healthcare needs must be respected and encouraged to ensure the continuous improvement of SUS services. Official surveys have been developed to assess the population’s perception of healthcare services, using validated instruments and structured questionnaires, such as the National Survey on Access, Use and Promotion of the Rational Use of Medicines (PNAUM) and the National Program for Improving Primary Care Access and Quality (PMAQ) [45,46]. However, most studies were published more than ten years ago [47,48,49,50,51,52,53]. These studies primarily involved the perceptions of users from a single municipality [51,52,54], and mainly utilized evaluation instruments that addressed only specific types of SUS services [16,31].

Given the scarcity of studies in Brazil and building evidence from across the country, a research project titled ‘*Assessment of Access and Quality of Public Health Services from the Perspective of the Unified Health System*’ was commissioned in 2023. This project is coordinated by a Professor at the Federal University of Rio de Janeiro (IPDG). To date, five regions of the State of Rio de Janeiro have participated, with approximately 1000 participants, resulting in publications covering the Coastal Lowland [55] and one municipality in the Northern Fluminense [56] regions. The recent publications [55,56] from different regions of the State of Rio de Janeiro (i.e., North Fluminense and Coastal Lowlands) have highlighted several challenges and weaknesses in pharmaceutical services. These issues stem primarily from a shortage of professionals available, in general, in less populated municipalities and fewer pharmacists working in public pharmacies, leading to consequences such as patients not receiving guidance and/or clarification on medication use from a pharmacist, as reported by several SUS users [55,56].

The present study focused on the Metropolitan region, which hosts the majority of the State of Rio de Janeiro’s population. The state capital has served as the federal district for many years, attracting investments and developments in healthcare within the city and its surroundings [57]. In a vast country such as Brazil with a universal healthcare system, it is crucial to undertake studies that assess, compare, and contrast user perceptions and identify weaknesses in different locations. Despite the expansion of the SUS, structural problems and disparities have worsened in recent years due to austerity measures [58]. As a result, there are significant regional disparities in access to healthcare services, which are associated with a higher percentage of Brazilians’ purchasing private health insurance and obtaining medication from private pharmacies. Given these regional disparities, initiatives that assess public perception across different regions of the country, and even within the same state, are particularly relevant, as they help to better understand the varying demands and experiences related to the SUS [58]. We define quality of care using a well recognized framework from 2000, which focuses on access and effectiveness in terms of whether people can get the care they need, and if the care is effective when they do. This framework also stresses the importance of user evaluation [59].

Consequently, the aim of this study was to assess users’ perceptions of the SUS regarding the quality of care in the Metropolitan Region of Rio de Janeiro, Brazil, which includes 19 municipalities. The results would subsequently enable a comparative analysis between the metropolitan region of Rio de Janeiro State and previously published studies [16,31,47,48,50,55,56] and, as a result, identify potential disparities and trends in access to and quality of health services across Brazil. As potential contributions and developments of this study, we highlight the dissemination of findings and the promotion of a broader discussion and debate on the identified weaknesses and challenges, particularly within spaces of social participation (e.g., health councils) and/or political arenas. Social participation is a key premise of the SUS [41], highlighting the importance of ongoing studies and initiatives aimed at understanding and valuing users’ perceptions. This is crucial for the independent health management process at all levels of coverage (federal, state, and municipal).

## 2. Methods

### 2.1. Study Design and Setting

A cross-sectional study was conducted to assess the perceptions of access to, and quality of, health services offered by the SUS from the perspective of residents in the Metropolitan Region of Rio de Janeiro State.

The state of Rio de Janeiro has a population of over 16 million inhabitants spread across 92 municipalities and the third highest average monthly per capita income among all the states in Brazil (BRL 2367.00–US$426.49) [60]. It is divided into eight regions, namely Metropolitan, Médio Paraíba, Central-South Fluminense, Mountain, Coastal Lowlands, Fluminense North, Fluminense Northwest, and Big Island Bay [57].

The Metropolitan Region encompasses 19 municipalities: Belford Roxo, Duque de Caxias, Itaguaí, Japeri, Magé, Mesquita, Nilópolis, Nova Iguaçu, Queimados, Rio de Janeiro, São João de Meriti, Seropédica, Itaboraí, Maricá, Niterói, Rio Bonito, São Gonçalo, Silva Jardim, and Tanguá [38,39,59]. Due to its economic development and extensive road and rail network, the Metropolitan Region attracts people from nearby regions, which also impacts healthcare services [38,39].

Of the 19 municipalities in the Metropolitan Region, three municipalities were included as a purposive convenience sample for the purpose of this study: the state capital (Rio de Janeiro), São Gonçalo, and Duque de Caxias. The three selected municipalities hold socioeconomic significance and are considered ‘reference’ points for providing a range of health services in this region of the State [61]. In addition, these three municipalities represent 68.7% of the total population of the Metropolitan Region. In this context, the city of Rio de Janeiro is the largest in the state considering both area and population, with 6,211,223 inhabitants and a per capita GDP of BRL 53,078.23 (US$9563.64) [60,62]. Its growing population generates a demand for housing, often resulting in poor living conditions, in addition to increasing unemployment [37]. The municipality of São Gonçalo has 896,744 inhabitants and a *per capita* GDP of R$18,504.81, while Duque de Caxias has 808,161 inhabitants and a *per capita* GDP of R$57,170.07 [60,62].

### 2.2. Data Collection Instrument

The questionnaire was interviewer-administered and was developed using, as a basis for question formulation, the questionnaires from projects conducted by the Ministry of Health. These include the National Survey on Access, Use, and Promotion of the Rational Use of Medicines (PNAUM) and the National Program for Improving Access and Quality of Primary Care (PMAQ) [45,46]. A pilot study was conducted in Macaé to better assess the understanding and comprehension of the instrument by the residents of this municipality. It must be noted that the questionnaire (Supplementary Material File S1) was the same questionnaire used in studies from the Mountain, Coastal Lowlands, North Fluminense, and Médio Paraíba regions [55,56].

The questionnaire developed by our project consisted of 66 questions, organized into four sections. These are (A) Socioeconomic profile and use of health services, including gender, age, education level, and household income; (B) Clinical conditions; (C) Use of medications; and (D) Perceptions and use of public health services. All participants answered the questions in sections A, B, and C, but only those who reported using the SUS completed Section D.

Section D focused on understanding participants’ views of access and the quality of public health SUS services. Questions were included about medication use and participants’ experiences regarding usage instructions and the role of the pharmacist, experiences in acquiring and using over-the-counter (OTC) medication and prescription medication, checking how they were used, and adherence to treatment recommendations. Additionally, questions were included about whether participants fully understood their prescriptions, for instance instructions regarding antibiotic use, concurrent alcohol consumption, and polypharmacy.

### 2.3. Data Collection and Inclusion Criteria

Data was collected between January and August 2024. A non-probabilistic sample size was calculated based on the minimum sample required to ensure a maximum margin of error of 7.0% in the estimation of overall percentages with 95% confidence, using the equation n = (1/d^2^). The total sample was distributed across the municipalities in proportion to their population sizes [63]. This is consistent with other studies within this research project [55,56].

Inclusion criteria for participants were individuals aged ≥18 years, who were approached through convenience sampling [64]. Some factors including the socioeconomic relevance of the selected municipalities, together with ease of access for data collectors and/or greater circulation of SUS users, coming from different social classes, involving the points of application of the questionnaire, were considered for the recruitment of participants. In addition, the number of inhabitants of each municipality were considered for the proportion of questionnaires distributed within each municipality covered by the study, applying the same data collection process as in our previous publications [55,56]. This process allowed for greater diversity in the sample. Data collection was conducted in central areas of the participating cities, particularly on streets and avenues with high pedestrian traffic and in proximity to public health facilities. This was undertaken by two undergraduate pharmacy students (M.C.R. and I.F.L.F.) from the School of Pharmaceutical Sciences at the Federal University of Rio de Janeiro/Macaé, who received prior data collection training from one of the authors.

Individuals (n = 200) who stated that they had never used SUS services were required to only answer the questions in sections A, B, and C. Section D was answered only by those who indicated in Sections A, B and C that they were using the health services offered by the SUS. Furthermore, individuals who reported exclusively purchasing medications from private pharmacies were excluded from questions about pharmaceutical services in the SUS.

The questionnaire was administered in Portuguese through interviews conducted in various public locations and freely accessible areas (such as public markets, squares, and avenues, among others). Participants were invited to participate voluntarily and were informed about the study’s objectives and contributions. Those who agreed to participate were asked to read and sign two copies of the Informed Consent Form, one to be kept by the participant and the other by the interviewer.

### 2.4. Data Analysis

Data analysis involved a descriptive analysis of the responses from participants, addressing sample characteristics including gender, age, education level, family income, clinical profile, and general use of public health services from the SUS, incorporating primary care, pharmaceutical services, and specialized services.

The evaluation focused on several key variables, including the profile of health service use (public only, private/health insurance only, or both), frequency and types of SUS services accessed, perceived relevance of the SUS, and users’ perceptions of access to and quality of public health services. Additionally, potential associations between participants’ gender, educational level, skin color, and family income, and their perceptions of access to and the quality of SUS services were explored. The evaluation also examined aspects related to the acquisition and use of medicines (public, private/health insurance, or both, including use without a prescription) and pharmaceutical services (e.g., presence and recognition of pharmacists in public pharmacies and the provision of guidance on medication use). In addition, we assessed users’ experiences within the pharmaceutical context, including the acquisition and use of both over-the-counter (OTC) and prescribed medicines, their adherence to prescribed treatments, and the challenges associated with polypharmacy. According to the WHO, polypharmacy is defined as the concurrent use of multiple medications, often referring to the regular use of five or more medications (including over-the-counter, prescription, and complementary medicines) [65].

Variables were summarized by absolute or relative frequency, and the association between them analyzed using the Pearson Chi-Square test, with results considered statistically significant if the *p*-value was <0.05. The analyses were performed using Microsoft Excel 365 and the R software version 4.3.0. The conversion rates used for the monetary values were provided by the Central Bank of Brazil (2024: USD 1 = BRL 5.55) [66].

### 2.5. Ethical Aspects

This study was approved by the Ethics Committee of the Federal University of Rio de Janeiro/Macaé Campus (CAAE: 68864623.6.0000.5699). Participants read and signed the consent forms before participation in the study.

## 3. Results

### 3.1. Population Characteristics

Data were collected from 200 participants in the city of Rio de Janeiro and from municipalities in the Metropolitan Region of Rio de Janeiro State. Of these participants, 78.5% were from the city of Rio de Janeiro, 11.3% from São Gonçalo, and 10.2% from Duque de Caxias. Overall, 54.5% were females, with 36.0% in the age range of 26 to 45 years. Among all participants, 86.0% reported using services offered by the SUS, and 29.0% had private health insurance. The remaining characteristics of the study population are summarized in Table 1.

**Figure 1:**
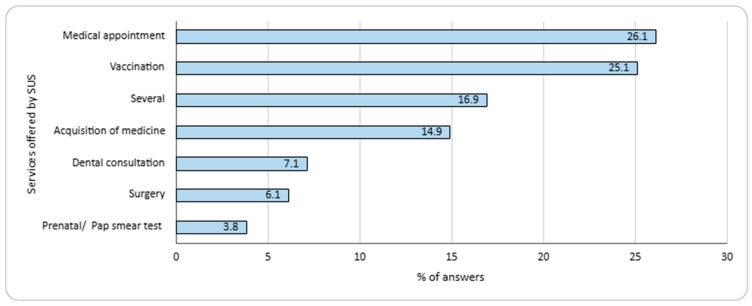
SUS services used by participants (n = 200). Participants could record more than one service.

The main clinical conditions reported by participants were hypertension (27.0%), anxiety (22.7%), dyslipidemia and diabetes mellitus (both conditions; 10.5%). However, 35.0% of participants stated they did not have any clinical conditions, and 31.5% had more than one condition. Among the responses, medical consultations were the most used SUS service (26.1%), followed by vaccinations (25.1%) and medications (16.9%), as shown in Figure 1. Among the main difficulties reported regarding medication use, the most notable were forgetfulness (22.3%), acquisition (17.0%), and polypharmacy (12.1%). Additionally, 5.3% of participants reported difficulties involving the need to avoid alcohol during a specific pharmacological treatment.

### 3.2. Responses from SUS Users

Overall, 196 (98.0%) participants subsequently proceeded to Section D of the questionnaire. When asked about the method of acquiring medications, 48.0% reported obtaining them privately from drugstores or private pharmacies, while 32.1% indicated acquiring medications both privately and through public SUS pharmacies. Only 14.3% of participants reported acquiring them exclusively for free through the SUS.

Considering that an individual with a health plan has the right and access to public SUS services, among those who reported having their medical consultations only in the private sector (n = 32), 75.0% had private health plans. However, the majority of participants in this profile use the SUS for this type of service, as well as for other services including vaccination. A third (32.3%) of those who reported using the vaccination service have private health insurance.

When asked about access to SUS services, those who reported using the SUS more frequently considered access to healthcare services to be better compared to those who said they used the services less frequently (*p* = 0.002), as showed in Table 2. Those who use the SUS more frequently also showed a positive trend (very good or good) regarding their perception of the quality of services, unlike those who reported using the services less often (*p* = 0.045). Additionally, 39.3% (n = 196) stated that they had never needed to leave their municipality to access SUS services, 33.8% of whom reported having no clinical conditions. Among those who stated they always had to leave the municipality to access SUS services, 33.3% reported having hypertension and 29.6% anxiety or depression.

Among our findings, we observed a tendency for participants with lower socioeconomic status to provide more favorable assessments of access to and quality of SUS services, as shown in Table 3. A statistically significant association was found between participants’ income and their perception of access to SUS services (*p* = 0.024). Among those earning less than one minimum wage, 83.3% rated access as very good or good. Conversely, 42.1% of those who rated access as bad or very bad had a household income above three minimum wages. A similar pattern was observed in the evaluation of service quality. Of participants earning less than one minimum wage, 76.7% rated the quality as very good or good, while 38.9% of those who rated it as bad or very bad had an income above three minimum wages.

Additionally, we highlight a trend toward a more favorable assessment of access to health services among male participants (*p* = 0.05) and those with Black skin color (*p* = 0.05). Regarding the evaluation of the quality of SUS services, a statistically significant association was observed only with the education variable, with participants with lower educational levels showing a greater tendency to rate the services more positively (*p* = 0.01).

Regarding medical appointments in the SUS, the process of scheduling appointments with a general practitioner scored a higher evaluation for access compared to accessing an appointment with a specialist. Table 4 shows that medical care in the SUS is perceived as very good/good by 62.6% of participants, while 37.4% expressed a negative and/or neutral perception of this type of service.

Participants indicated that the most important factors regarding the quality of health services are the geographic accessibility of the service location (50.5%) and the ease of scheduling appointments (46.9%). In addition, the three factors indicated by the participants as the most important to be improved in the SUS were the infrastructure (48.5%), associated with the need for a greater number of establishments to provide public health services, such as basic health units, and/or the improvement of the conditions of the facilities already available to users, the qualification of professionals (30.6%), and easier access to medicines (16.8%).

Among the 196 participants who used the SUS, 92 (46.9%) indicated that they obtain medications from public services, as showed in (Table 5). These participants were asked about their perception of the dispensing units and the role of the pharmacist. The results showed that 48.3% of patients always obtained the medications they needed in public pharmacies and 38.9% stated that they always receive instructions on how to use their medications. Almost half (48.1%) also reported the presence of a pharmacist or another staff member always being available to answer their questions. However, 49.4% of participants reported that when their medicines were dispensed, they never received information on the correct way to store them.

When asked about their knowledge of the pharmaceutical services provided, only 24.0% of the participants answered that they know what services can be provided, and 37.0% answered that they do not know or chose not to respond to this question (n = 92).

## 4. Discussion

This paper presents the findings of the fourth study in an ongoing research program aimed at capturing the perspectives of SUS users in Brazil. In the 2019 National Health Survey [67], it was observed that 35% of the population in the state of Rio de Janeiro had private health insurance. The results in this study indicate that the majority of participants rely on the SUS, with 29.0% having health insurance, a percentage similar to that observed in other regions of Rio de Janeiro State [55,56] and the national scenario reported by ANS [35]. Consequently, most of the population report depending exclusively on the public SUS system, underscoring the importance of studies that emphasize locally responsive community participation in the continuous improvement of this universal healthcare system across Brazil.

A significant number of participants reported positive perceptions and/or ease of access to services, such as specialist appointments in this region. This contrasts with findings from other studies conducted in the interior of the same state, specifically in the Coastal Lowlands [55] and North Fluminense [56] regions, which are part of this broader research project. Among the most frequent SUS users in our study, 68.2% rated access to services as very good or good, whereas 33.9% of those who use the SUS less frequently rated access as very poor (*p*-value = 0.002). In a similar evaluation conducted in another region of Rio de Janeiro, the Coastal Lowlands, 40.0% of SUS users who always relied on the system considered access to be neither good nor bad, while another 40.0% rated it as very good or good [55]. Differences in access perceptions have also been observed across various regions of the state [55,56].

These findings align with the existing literature, which suggests that better access to services is often correlated with factors such as being in state capitals, larger municipalities, and having a higher number of healthcare teams per basic health unit [68]. This may explain the more favorable results observed in our study, given that it focused on a metropolitan region. Additionally, we believe that users who rely on SUS services more frequently tend to have a better understanding of the processes and procedures involved in accessing different types of healthcare services, which may facilitate their navigation through the system. In this context, we highlight that some studies suggest that a user’s lack of knowledge and experience with the system can hinder their access to healthcare services and limit public participation in healthcare management [51,52].

Furthermore, 39.3% of SUS users in the Metropolitan Region reported that they had never needed to leave their municipality to access healthcare services. Among them, 33.8% stated that they had no clinical conditions. It is worth noting that the lower need to seek care in other municipalities may be related to the fact that a large proportion of the respondents (approximately 52.5%) were between 26 and 56 years old and presented clinical conditions (e.g., hypertension, anxiety, or depression) that are typically managed within primary care settings such as basic health units. Additionally, the municipalities included in this study, particularly those located in the metropolitan region of one of Brazil’s largest states, benefit from a more extensive network of healthcare services and facilities. This may provide residents with easier access to healthcare services compared to those living in more rural or interior areas of the state [55,56], as observed in another study published by our research group as part of this larger project. It is important to emphasize that metropolitan regions generally offer a greater number and variety of healthcare services, which may contribute to better access and convenience for local populations [57,68]. This finding aligns with the study by Souza et al. (2024) in the Coastal Lowlands region, where patients often needed to travel to other municipalities to obtain specialist consultations [55]. The Metropolitan Region continues to be a key focus for healthcare investment and development, maintaining a degree of connectivity with other regions [57].

Among participants who reported always using SUS services, 60.4% had a positive perception of service quality, rating it as very good or good. In contrast, those who used the system less frequently tended to rate the quality as poor or very poor (*p* = 0.045). This result is consistent with previous research, including the survey conducted by the Institute of Applied Economic Research (IPEA) in 2011 and the National Health Survey by IBGE in 2019 [50,69]. In the socioeconomic context, an evaluation of the correlation between participants’ income and their perceptions of access (*p* = 0.024) and quality (*p* = 0.149) revealed that individuals with lower incomes tend to rate access to public health services more positively. This may be attributed to the fact that lower-income individuals typically use SUS services more frequently and rely on a wider range of services, with the majority being fully dependent on the public healthcare system [50,69].

The most frequently used SUS service among participants was the scheduling of medical appointments, reported by 26.1% of respondents. Only 38.2% of participants rated the process of scheduling appointments with a general practitioner as very good or good, while a similar proportion (38.7%) rated it as poor or very poor. This suggests that many users face difficulties in accessing this type of service in the study areas. Similarly, for specialist appointments, only 33.4% of participants rated the scheduling process as very good or good, whereas 41.3% rated it as poor or very poor. These findings are consistent with previous studies conducted in the state of Rio de Janeiro, which also reported user dissatisfaction and concerns related to the scheduling of medical and specialist appointments within the SUS [55,56]. Such issues remain a challenge in the context of health system management in these regions, particularly when analyzed comparatively. We believe that these results may contribute to stimulating discussions in forums such as health councils, supporting dialogue and the planning of actions aimed at better addressing community needs. It is important to note that these challenges are not exclusive to low- and middle-income countries (LMICs) implementing universal healthcare. Similar difficulties and dissatisfaction with appointment scheduling processes have also been reported in high-income countries such as Canada and the United Kingdom [70,71].

Regarding public pharmaceutical services, one of the main challenges reported by participants was the difficulty in acquiring medications. Among the key issues identified during the interviews were bureaucratic requirements such as prescriptions needing specific documentation from prescribers, which were not always issued promptly, as well as frequent delays in medication availability. As a result, although many users rely on the SUS for services such as medical consultations and vaccinations, 48.0% stated that they obtain their medications exclusively from private pharmacies. Among those who do access public pharmacies, only 48.3% reported always found the medicines they needed, often having to wait for restocking. This situation reflects a broader shift in the role of the state in pharmaceutical provision, from a unified system of free distribution to a complementary public–private model with user co-payment [27], underscoring the underfunding of the SUS. Boing et al. (2022) also emphasized persistent barriers to accessing medications through the SUS, along with a rise in out-of-pocket spending, which disproportionately affects lower-income populations dependent on these services [28].

Among the 200 participants surveyed, 29.5% reported never having received any guidance from a pharmacist regarding their medications. Additionally, 42.3% of those who obtained medicines from public pharmacies stated that they had never encountered a pharmacist at the health facility. These findings are consistent with data from the 2015 PNAUM, which showed that only 44.5% of primary care dispensing units had a pharmacist present during operating hours, most of whom were engaged in logistical tasks rather than direct patient care [15]. The results related to medicine access and pharmacist involvement also align with findings by Viana et al., who noted that budget cuts to programs such as *Farmácia Popular* and *Garantia da Assistência Farmacêutica* in Rio de Janeiro between 2015 and 2018 negatively impacted the availability and quality of pharmaceutical services [72]. Similar issues were also observed in other studies from our research project [55,56], particularly the shortage of pharmacists dedicated to delivering pharmaceutical care and individualized therapeutic follow-up. Pharmacists in primary care settings play a fundamental role in promoting the rational use of medications, supporting health education, reviewing prescriptions, counseling patients and families, and conducting pharmacotherapeutic monitoring [13,16,31,73]. We believe that the findings from this and other studies may help stimulate discussions within professional councils about expanding the role and presence of pharmacists in Primary Health Care. Raising awareness among healthcare managers about the value of pharmacists is essential as they are key members of multidisciplinary teams and crucial to optimizing pharmacotherapy. Encouragingly, some efforts have now been made including increased hiring and ongoing dialogue about the relevance of pharmacists in primary care [74].

We acknowledge that this study had some limitations. Of the nineteen municipalities in the region, only three were included. However, these are among the most populous and socioeconomically significant [57,60,62]. While the participant profile showed similarities to the overall population of the Metropolitan Region of Rio de Janeiro State, the use of convenience sampling where participants were selected based on availability and accessibility may have limited the ability to identify broader patterns and achieve full representation of the wider population, which can lead to a selection bias. Furthermore, rural areas were not involved in the municipalities included in this study. However, we believe a wide variety of opinions and profiles were included in the study. In addition, there is the possibility of recall bias among participants, with these biases potentially impacting the interpretation of the study findings. It is important to note that no analysis was conducted regarding the association between age and participants’ perceptions of access to and quality of SUS health services. Our analyses focused on skin color, gender, and family income. Despite these limitations, we believe that the results can contribute to the current understanding of the perceptions of SUS users in the Metropolitan Region of Rio de Janeiro. We emphasize that this study focused on one of the main regions of the state of Rio de Janeiro, as part of a research project that evaluated a total of five regions and more than 1000 interviewees from the same state. Based on this methodological strategy, we believe that assessments in different regions of the same state can contribute to discussions on the management of the SUS in an important state, particularly from the socioeconomic perspective of the country.

While the study used a non-probability and convenience sample, it provides a foundation for practical improvements in public health services, particularly within the Brazilian Unified Health System (SUS). The findings offer valuable insights into users’ experiences that can support more informed decision-making by health managers. These results highlight the need for targeted investments in underserved areas, improved coordination within health regions, and the development of planning strategies that prioritize equity and efficiency in service delivery. Improved coordination can be achieved by strengthening Regional Inter-Management Committees (CIRs), integrating health information systems across municipalities, and promoting joint regional planning based on shared health indicators. Planning strategies that prioritize equity and efficiency should include evidence-based resource allocation, the identification of service delivery gaps, a continuous monitoring of access and performance indicators, and the active engagement of civil society in defining local priorities. By fostering discussions on regionalization, resource distribution, and user satisfaction, the study contributes to the strengthening of public health policy and management practices in Brazil. Furthermore, the lessons drawn may inform similar efforts in other low-and middle-income countries (LMICs) pursuing universal health coverage.

## 5. Conclusions

This study is part of a series of an ongoing program analyzing perceptions of access to and the quality of SUS services in different regions of the state of Rio de Janeiro. Unfortunately, there is no large-scale assessment of this kind in the country, which may be due to logistical and financial challenges inherent to a country of continental dimensions. Nevertheless, such strategies can help promote and emphasize the importance of initiatives that foster social participation, particularly by enabling users to express their perceptions of the various services offered by the SUS. The main findings indicate that those who use the system more frequently tend to rate access and quality more positively. This pattern has been observed in our previous publications within a project assessing access to and the quality of public health services in the state of Rio de Janeiro. We emphasize that, as a metropolitan region typically recognized for offering a greater number of health facilities at various levels of complexity, its residents generally report better quality and availability of services compared to users in the interior regions of the state.

Through a series of studies [16,31,34,35,49,50,52], we have identified that some challenges are commonly cited in study settings across Brazil, including infrastructure issues, the scheduling process for specialist appointments, and the availability of pharmacists within healthcare units. Additionally, the main challenges regarding medication use include forgetfulness and difficulties with access or purchasing medications. There are also concerns about the limited number of patients receiving proper instructions on how to use their medications. Pharmacists are not considered mandatory members of the minimum team in basic health units within the PHC framework in both the state of Rio de Janeiro and throughout Brazil. In addition, key areas for improvement within the SUS include the role of pharmacists, and we emphasize the importance of ongoing discussions involving regional pharmacy councils and healthcare managers about the contributions and potential impact of pharmacists in multidisciplinary teams and their effect on SUS users.

Finally, our findings highlight the growing need for studies that stimulate discussions and reflections on healthcare needs, aiming to support a more strategic reassessment of policies and actions to better serve the population in this region. We stress that each region within a state or country has its own socioeconomic characteristics, which shape diverse healthcare needs. This underscores the necessity of conducting studies that contribute to assessments and, more importantly, disseminate users’ perceptions of a universal healthcare system that is continuously evolving to better serve its population.

## Figures and Tables

**Table 1:** Characteristics of study participants (n = 200).

Variable	n	(%)
Gender		
Female	109	54.5%
Male	87	43.5%
Other	4	2.0%
Age Profile (years)		
18–25	36	18.0%
26–45	72	36.0%
46–60	53	26.5%
More than 60	39	19.5%
Skin color		
White	74	37.0%
Black	48	24.0%
Brown	60	30.0%
Other	18	9.0%
Education level *		
Never attended school	1	0.5%
Incomplete elementary school	26	13.0%
Completed elementary school	14	7.0%
Incomplete high school	12	6.0%
Completed high school	65	32.5%
Incomplete college	33	16.5%
Completed college or more	24	12.0%
Family Income ** (Number of times the minimum wage ***)		
Up to 1	31	15.5%
1–2	34	17.0%
2–3	20	10.0%
3–5	25	12.5%
5–10	9	4.5%
10–20	3	1.5%
>20	0	0.0%
Use of SUS services—Yes	**172**	**86.0%**
Has a private health plan—Yes	**58**	**29.0%**

Notes: * Education level: 12% of participants did not answer these questions (“don’t know/don’t want to answer”); ** Family income: 39% of participants did not answer these questions (“don’t know/don’t want to answer”); *** Minimum wage in 2024: BRL 1412.00 (US$254.41).

**Table 2:** Views of SUS users regarding the access to and quality of the public health services associated with frequency (n = 196).

Access to SUS Services n (%)				
Frequency	Very Good/Good	Neither Good nor Bad	Bad/Very Bad	*p*-Value
Always	58 (68.2%)	15 (17.7)	12 (14.1%)	0.002
Frequently	19 (57.6%)	11 (33.3%)	3 (9.1%)	
Sometimes/Rarely/ Never	23 (40.0%)	16 (27.1%)	20 (33.9%)	
TOTAL *	100 (56.5%)	42 (23.7%)	35 (19.8%)	
**Quality of SUS services n (%)**				
**Frequency**	**Very Good/Good**	**Neither Good nor Bad**	**Bad/Very Bad**	***p*-Value**
Always	55 (60.4%)	23 (25.3%)	13 (14.3%)	
Frequently	19 (55.9%)	11 (32.3%)	4 (11.8%)	
Sometimes/Rarely/ Never	25 (41.7%)	16 (26.7%)	19 (31.6%)	0.045
TOTAL **	99 (53.5%)	50 (27.0%)	36 (19.5%)	

Notes: * 9.7% of participants did not answer these questions (“don’t know/don’t want to answer”); ** 5.6% of participants did not answer these questions (“don’t know/don’t want to answer”).

**Table 3:** Views of SUS users regarding the access to and quality of the public health services associated with family income (n = 196).

Access to SUS Services n (%)				
Family Income (Number of Times the Minimum Wage)	Very Good/Good	Neither Good nor Bad	Bad/Very Bad	*p*-Value
Up to 1	25 (83.3%)	3 (10.0%)	2 (6.7%)	
1–2	19 (57.6%)	8 (24.2%)	6 (18.2%)	
2–3	8 (47.1%)	6 (35.3%)	3 (17.6%)	
>3	11 (36.7%)	11 (36.7%)	8 (26.6%)	0.024
TOTAL *	63 (57. 3%)	28 (25.4%)	19 (17.3%)	
**Quality of SUS services n (%)**				
**Family Income (Number of Times the Minimum Wage)**	**Very Good/Good**	**Neither Good nor Bad**	**Bad/Very Bad**	***p*-Value**
Up to 1	23 (76.7%)	5 (16.7%)	2 (6.6%)	
1–2	17 (51.5%)	11 (33.3%)	5 (15.2%)	
2–3	9 (45.0%)	7 (35.0%)	4 (20,0%)	0.149
>3	13 (40.6%)	12 (37.5%)	7 (21.9%)	
TOTAL **	62 (53. 9%)	35 (30.4%)	18 (15.7%)	

Notes: * 43.9% of participants did not answer these questions (“don’t know/don’t want to answer”); ** 41.3% of participants did not answer these questions (“don’t know/don’t want to answer”).

**Table 4:** Views of SUS users regarding medical consultations of the public health services (n = 196).

Questions	Answers n (%)					
	Very Good	Good	Nor Good or Bad	Bad	Very Bad	Total
How do you consider the process for marking medical consultations with the general practitioner through the SUS? *	14 (8.1%)	52 (30.1%)	40 (23.1%)	35 (20.2%)	32 (18.5%)	173 (100.0%)
How do you consider the process for marking medical consultations with specialists through the SUS? **	10 (6.2%)	44 (27.2%)	41 (25.3%)	26 (16.0%)	41 (25.3%)	162 (100.0%)
How do you consider the medical care offered by the SUS? ***	33 (18.4%)	79 (44.1%)	45 (25.1%)	9 (5.0%)	13 (7.4%)	179 (100.0%)

Notes: * 11.7% of participants did not answer this question (“don’t know/don’t want to answer”); ** 17.3% of participants did not answer this question (“don’t know/don’t want to answer”); *** 8.7% of participants did not answer this question (“don’t know/don’t want to answer”).

**Table 5:** Views of SUS users regarding pharmaceutical services of the public health services (n = 196).

Questions	Answers n (%)					
	Always	Often	Sometimes	Rarely	Never	Total
In the last three months, how often did you get the medicines you were looking for in public pharmacies? *	42 (48.3%)	17 (19.5%)	20 (23.0%)	4 (4.6%)	4 (4.6%)	87 (100.0%)
When collecting medicines from the SUS pharmacy, do the employees who deliver them provide instructions on how to use them? **	35 (38.9%)	17 (18.9%)	11 (12.2%)	8 (8.9%)	19 (21.1%)	90 (100.0%)
Do you receive information on how to store medicines at home? ***	18 (20.7%)	7 (8.1%)	15 (17.2%)	4 (4.6%)	43 (49.4%)	87 (100.0%)
Have you already met the pharmaceutical professional at the basic health unit you frequent? ****	14 (17.9%)	18 (23.1%)	5 (6.4%)	8 (10.3%)	33 (42.3%)	78 (100.0%)
Is the pharmacist or other employees available to answer questions about the medicines? *****	39 (48.2%)	15 (18.5%)	15 (18.5%)	2 (2.5%)	10 (12.3%)	81 (100.0%)

Notes: * 5.4% of participants did not answer this question (“don’t know/don’t want to answer”); ** 2.2% of participants did not answer this question (“don’t know/don’t want to answer”); *** 5.4% of participants did not answer this question (“don’t know/don’t want to answer”); **** 15.2% of participants did not answer this question (“don’t know/don’t want to answer”); ***** 12.0% of participants did not answer this question (“don’t know/don’t want to answer”).

## Data Availability

The data presented in this study are available on request from the corresponding author. The data are not publicly available due to privacy and ethical restrictions.

## Supplementary Materials

The following supporting information can be downloaded at: https://www.mdpi.com/article/10.3390/ijerph22060967/s1, Survey Instrument S1.

## References

[1] Brasil (1988). Constituição da República Federativa do Brasil.

[2] Brasil (1990). Lei nº 8.080, de 19 de setembro de 1990. Dispõe Sobre as Condições para a Promoção, Proteção e Recuperação da Saúde, a Organização e o Funcionamento dos Serviços Correspondentes e dá Outras Providências.

[3] Ministério da Saúde (2011). Carta dos Direitos dos Usuários da Saúde.

[4] Castro M.C., Massuda A., Almeida G., Menezes-Filho N.A., Andrade M.V., de Souza Noronha K.V.M., Rocha R., Macinko J., Hone T., Tasca R. (2019). Brazil’s unified health system: The first 30 years and prospects for the future. Lancet.

[5] Brasil (2000). Emenda Constitucional nº 29, de 13 de Setembro de 2000. Constituição da República Federativa do Brasil de 1988: Altera os arts. 34, 35, 156, 160, 167 e 198 da Constituição Federal e Acrescenta Artigo ao Ato das Disposições Constitucionais Transitórias, para Assegurar os Recursos Mínimos para o Financiamento das Ações e Serviços Públicos de Saúde.

[6] Brasil. Ministério da Saúde (2010). Portaria Nº 4.279, de 30 de Dezembro de 2010. Estabelece Diretrizes para a Organização da Rede de Atenção à Saúde no Âmbito do Sistema Único de Saúde (SUS).

[7] Gomes R.M., Barbosa W.B., Godman B., Costa J.D.O., Ribeiro Junior N.G., Simão Filho C., Cherchiglia M.L., Acurcio F.D.A., Guerra Junior A.A. (2020). Effectiveness of maintenance immunosuppression therapies in a matched-pair analysis cohort of 16 years of renal transplant in the Brazilian National Health System. Int. J. Environ. Res. Public Health.

[8] Brasil. Ministério da Saúde Atenção Primária e Atenção Especializada: Conheça os Níveis de Assistência do Maior Sistema Público de Saúde do Mundo. https://www.gov.br/saude/pt-br/assuntos/noticias/2022/marco/atencao-primaria-e-atencao-especializada-conheca-os-niveis-de-assistencia-do-maior-sistema-publico-de-saude-do-mundo.

[9] Brasil. Ministério da Saúde (1997). Saúde da Família: Uma Estratégia para a Reorganização do Modelo Assistencial.

[10] Andrade M.V., Coelho A.Q., Neto M.X., de Carvalho L.R., Atun R., Castro M.C. (2018). Brazil’s Family Health Strategy: Factors associated with programme uptake and coverage expansion over 15 years (1998–2012). Health Policy Plan..

[11] Starfield B., Shi L., Macinko J. (2005). Contribution of primary care to health systems and health. Milbank Q..

[12] World Health Organization, Organization for Economic Co-operation and Development, International Bank for Reconstruction and Development (2018). Delivering Quality Health Services: A Global Imperative for Universal Health Coverage.

[13] Brasil. Ministério da Saúde (2023). Portaria GM/MS nº 635, de 22 de maio de 2023. Institui, Define e Cria Incentivo Financeiro Federal de Implantação, Custeio e Desempenho para as Modalidades de Equipes Multiprofissionais na Atenção Primária à Saúde.

[14] Brasil. Ministério da Saúde Estratégia Saúde da Família. https://www.gov.br/saude/pt-br/composicao/saps/esf.

[15] Brasil. Ministério da Saúde (2017). Secretaria de Ciência, Tecnologia e Insumos Estratégicos. Componente Avaliação dos Serviços de Assistência Farmacêutica Básica: Resultados.

[16] Brasil. Ministério da Saúde (2012). Portaria nº 2.077, de 17 de Setembro de 2012. Institui a Pesquisa Nacional Sobre Acesso, Utilização e Promoção do Uso Racional de Medicamentos no Brasil (PNAUM).

[17] Brasil. Ministério da Saúde Componente Básico da Assistência Farmacêutica (CBAF). https://www.gov.br/saude/pt-br/composicao/sectics/daf/cbaf.

[18] Brasil. Ministério da Saúde Componente Estratégico da Assistência Farmacêutica (CESAF). https://www.gov.br/saude/pt-br/composicao/sectics/daf/cesaf.

[19] Brasil. Ministério da Saúde Componente Especializado da Assistência Farmacêutica (CEAF). https://www.gov.br/saude/pt-br/composicao/sectics/daf/ceaf.

[20] Brasil. Ministério da Saúde (2016). Portaria nº 111, de 28 de Janeiro de 2016. Dispõe Sobre o Programa Farmácia Popular do Brasil (PFPB).

[21] Barbosa M.M., Nascimento R.C., Garcia M., Acurcio F.A., Godman B., Guerra A.A., Alvares-Teodoro J. (2021). Strategies to improve the availability of medicines in primary health care in Brazil: Findings and implications. J. Comp. Eff. Res..

[22] Brasil. Ministério da Saúde Programa Nacional de Imunizações—Vacinação. https://www.gov.br/saude/pt-br/acesso-a-informacao/acoes-e-programas/pni.

[23] Fernandes C.M., Dias S.L., Ferreira M.C., Luna E.J.A. (2021). COVID-19 post-vaccination in healthcare workers and vaccine effectiveness. Clinics.

[24] Paixao E.S., Wong K.L.M., Alves F.J.O., de Araújo Oliveira V., Cerqueira-Silva T., Júnior J.B., Machado T.M., Junior E.P.P., Boaventura V.S., Penna G.O. (2022). CoronaVac vaccine is effective in preventing symptomatic and severe COVID-19 in pregnant women in Brazil: A test-negative case-control study. BMC Med..

[25] Zheng C., Shao W., Chen X., Zhang B., Wang G., Zhang W. (2021). Real-world effectiveness of COVID-19 vaccines: A literature review and meta-analysis. Int. J. Infect. Dis..

[26] Brasil. Ministério da Saúde Assistência Farmacêutica e Insumos Estratégicos: Sobre o DAF. https://www.gov.br/saude/pt-br/composicao/sectics/daf.

[27] Hasenclever L., Miranda C., Chaves G., Peixoto A.L.A., Mattos L.V., Viana J.S. (2021). Controversial aspects of the concept of health needs and their impact on the accessibility of medicine and health services. Cien. Saude Colet..

[28] Boing A., Andrade F., Bertoldi A., Peres K., Massuda A., Boing A. (2022). Prevalências e desigualdades no acesso aos medicamentos por usuários do Sistema Único de Saúde no Brasil em 2013 e 2019. Cad. Saude Publica.

[29] Conselho Federal de Farmácia Nossos dados. https://site.cff.org.br/estatistica.

[30] Faraco E.B., Guimarães L., Anderson C., Leite S.N. (2020). The pharmacy workforce in public primary healthcare centers: Promoting access and information on medicines. Pharm. Pract..

[31] Peixoto R.T., Campos M.R., Luiza V.L., Mendes L.V. (2022). O farmacêutico na atenção primária à saúde no Brasil: Análise comparativa 2014–2017. Saúde Debate.

[32] Brasil. Ministério da Saúde (2008). Portaria n° 154, de 24 de Janeiro de 2008. Cria os Núcleos de Apoio à Saúde da Família—NASF. Diário Oficial da União.

[33] Brasil. Ministério da Saúde Equipe de Saúde da Família. https://www.gov.br/saude/pt-br/composicao/saps/esf/equipe-saude-da-familia.

[34] Cadastro Nacional de Estabelecimentos de Saúde (CNES), Federação Brasileira de Hospitais (2022). Cenário dos Hospitais no Brasil 2021–2022.

[35] Brasil. Ministério da Saúde. Agência Nacional de Saúde Suplementar Setor de Planos de Saúde Fecha 2024 com Números Recordes de Beneficiários. https://www.gov.br/ans/pt-br/assuntos/noticias/numeros-do-setor/setor-de-planos-de-saude-fecha-2024-com-numeros-recordes-de-beneficiarios.

[36] de Jesus W.L., Assis M.M. (2010). Systematic review about the concept of access to health services: Planning contributions. Cien. Saude Colet..

[37] Brasil. Ministério da Saúde. Regiões de Saúde https://www.gov.br/saude/pt-br/composicao/saps/programa-cuida-mais-brasil/regioes-de-saude.

[38] Governo do Estado do Rio de Janeiro (2020). Diagnóstico de Saúde da Região Metropolitana I.

[39] Governo do Estado do Rio de Janeiro (2020). Diagnóstico de Saúde da Região Metropolitana II.

[40] Casa Fluminense Mapa da Desigualdade 2020: Região Metropolitana do Rio de Janeiro. https://www.casafluminense.org.br/wp-content/uploads/2020/07/mapa-da-desigualdade-2020-final_compressed.pdf.

[41] Brasil (1990). Lei nº 8.142, de 28 de Dezembro de 1990. Dispõe Sobre a Participação da Comunidade na Gestão do Sistema Único de Saúde (SUS) e Sobre as Transferências Intergovernamentais de Recursos Financeiros na Área da Saúde e dá Outras Providências.

[42] Silva R.C.C., de Novais M.A.P., Zucchi P. (2021). Social participation in the unified health system of Brazil: An exploratory study on the adequacy of health councils to resolution 453/2012. BMC Health Serv. Res..

[43] Santos C.L., Santos P.M., Pessali H.F., Rover A.J. (2020). Health councils and dissemination of SUS management instruments: An analysis of portals in Brazilian capitals. Cien. Saude Colet..

[44] Koler J., Martinez M.G. (2015). Participatory health councils and good governance: Healthy democracy in Brazil?. Int. J. Equity Health.

[45] Brasil. Ministério da Saúde (2016). Secretaria de Ciência, Tecnologia e Insumos Estratégicos. Componente Populacional: Introdução, Método e Instrumentos.

[46] Brasil. Ministério da Saúde (2015). Portaria nº 1.645, de 2 de Outubro de 2015. Dispõe Sobre o Programa Nacional de Melhoria do Acesso e da Qualidade da Atenção Básica (PMAQ-AB).

[47] Protásio A.P.L., Gomes L.B., Machado Ldos S., Valença A.M.G. (2017). Satisfação do usuário da atenção básica em saúde por regiões do Brasil: 1º ciclo de avaliação externa do PMAQ-AB. Cien. Saude Colet..

[48] Lima J.G., Giovanella L., Fausto M.C.R., Bousquat A., da Silva E.V. (2018). Atributos essenciais da atenção primária à saúde: Resultados nacionais do PMAQ-AB. Saude Debate.

[49] Abreu D.M.X., Araújo L.H.L., Reis C.M.R.D., Lima Â.M.L.D., Santos A.F.D., Jorge A.O., Fonseca Sobrinho D., Machado A.T.G.D.M. (2018). Service users’ perception about healthcare provided by teams participating in the National Program for Primary Care Access and Quality Improvement in Brazil. Epidemiol. Serv. Saude.

[50] Instituto de Pesquisa Econômica Aplicada (IPEA) Sistema de Indicadores de Percepção Social (SIPS). https://www.ipea.gov.br/portal/images/stories/PDFs/SIPS/110207_sipssaude.pdf.

[51] Fonseca G.S., Paulino T.S.C., Morais I.F., Valença C.N., Germano R.M. (2013). Percepção de usuários e profissionais de saúde sobre o sistema único de saúde no município de Santa Cruz-RN. Rev. Bras. Promoc. Saude.

[52] Hamada R.K.F., Almeida V.O.C., Brasil I.C.F., Souza S.G., Luzia R.A.B., Campos E.M.S., Leite I.C.G. (2020). Conhecendo o Sistema Único de Saúde: Um olhar da população. Rev. APS.

[53] Szwarcwald C.L., Damacena G.N., Souza Júnior P.R., Almeida W.S., Malta D.C. (2016). Perception of the Brazilian population on medical health care. Brazil, 2013. Cien. Saude Colet..

[54] Oliveira G.L., Xavier C.C., Ribeiro A.P., Proietti F.A. (2022). Users’ perception about primary care attributes in a metropolitan region of Minas Gerais. APS Rev..

[55] Souza J.S., Reis E.A., Godman B., Campbell S.M., Meyer J.C., Sena L.W.P., Godói I.P.D. (2024). Users’ perceptions of access to and quality of Unified Health System services in Brazil: A cross-sectional study and implications to healthcare management challenges. Int. J. Environ. Res. Public Health.

[56] Dornas B.S.S., Reis E.A., Campos A.A.O., Godói I.P.D. (2024). Percepções sobre os serviços de saúde pública em Macaé-RJ: Um estudo transversal. Rev. Saúde Pública Paraná.

[57] Kuschnir R., Chorny A., Lira A.M.L., Sonoda G., Fonseca T.M.P., Ugá M.A.D., Martins M., Braga Neto F.C. (2010). Regionalização no estado do Rio de Janeiro: O desafio de aumentar acesso e diminuir desigualdades. A Gestão do Sus no Âmbito Estadual: O Caso do Rio de Janeiro.

[58] Massuda A., Hone T., Leles F.A.G., Castro M.C., Atun R. (2018). The Brazilian health system at crossroads: Progress, crisis and resilience. BMJ Glob. Health.

[59] Campbell S.M., Roland M.O., Buetow S.A. (2000). Defining quality of care. Soc. Sci. Med..

[60] Instituto Brasileiro de Geografia e Estatística (IBGE) Censo Demográfico Brasileiro de 2022. Rio de Janeiro, Brazil, 2022. https://www.ibge.gov.br/estatisticas/sociais/populacao/22827-censo-demografico-2022.html?edicao=35938&t=resultados.

[61] Secretaria de Estado da Casa Civil e Desenvolvimento Econômico do Rio de Janeiro (2017). Caderno Regional do Estado do Rio de Janeiro: Região Metropolitana Desenvolvimento Socioeconômico 2007/2014. https://www.gov.br/empresas-e-negocios/pt-br/portais-desconhecidos/observatorioapl/biblioteca-apl/publicacoes/caderno-regional-metropolitana-rj.pdf.

[62] IBGE—Instituto Brasileiro de Geografia e Estatística Produto Interno Bruto dos Municípios. https://www.ibge.gov.br/estatisticas/economicas/contas-nacionais/9088-produto-interno-bruto-dos-municipios.html?t=pib-por-municipio&c=1100031.

[63] Bolfarine H., Bussab W.O. (2005). Elementos de Amostragem.

[64] Galloway A., Kempf-Leonard K. (2005). Non-Probability Sampling. Encyclopedia of Social Measurement.

[65] World Health Organization (2019). Medication Safety in Polypharmacy.

[66] Brasil (2024). Histórico de Cotação.

[67] IBGE—Agência de Notícias Instituto Brasileiro de Geografia e Estatística Pesquisa Nacional de Saúde 2019: Informações sobre Domicílios, Acesso e Utilização dos Serviços de Saúde. Rio de Janeiro, 2020. https://biblioteca.ibge.gov.br/visualizacao/livros/liv101748.pdf.

[68] Alves M.G.M., Casotti E., Oliveira L.G.D., Machado M.T.C., Almeida P.F., Corvino M.P.F., Marin J., Flauzino R.F., Montenegro L.A.A. (2014). Factors affecting access to the Family Health Strategy teams in Brazil. Saude Debate.

[69] IBGE—Agência de Notícias Instituto Brasileiro de Geografia e Estatística Pesquisa Nacional de Saúde 2019: Atenção Primária à Saúde e Informações Antropométricas. Rio de Janeiro, 2021. https://www.pns.icict.fiocruz.br/wp-content/uploads/2021/02/liv101758.pdf.

[70] Angus Reid Institute (2023). CMA Report: Public Opinion on Healthcare in Canada. https://angusreid.org/wp-content/uploads/2023/08/2023.08.17_CMA.pdf.

[71] NHS—National Health Service Understanding Public Perceptions and Attitudes to the NHS. NHS Confederation 2023. https://www.nhsconfed.org/publications/understanding-public-perceptions-and-attitudes-nhs.

[72] Viana L.F., Silva G.H., Godói I.P.D. (2024). Financiamento da saúde pública no estado do Rio de Janeiro: Panorama (2015–2018), desafios e reflexões. J. Assist. Farm. Farm..

[73] Costa K.S., Tavares N.U.L., Nascimento Júnior J.M.d., Mengue S.S., Álvares J., Junior A.A.G., de Assis Acurcio F., Soeiro O.M. (2017). Pharmaceutical services in the primary health care of the Brazilian Unified Health System: Advances and challenges. Rev. Saude Publica.

[74] Conselho Federal de Farmácia (2023). XXXVII Conasems: CFF Debate Papel do Farmacêutico na Atenção Primária. https://site.cff.org.br/noticia/noticias-do-cff/21/07/2023/xxxvii-conasems-cff-debate-papel-do-farmaceutico-na-atencao-primaria.

